# Impact of organizational culture, occupational commitment and industry-academy cooperation on vocational education in China: Cross-sectional Hierarchical Linear Modeling analysis

**DOI:** 10.1371/journal.pone.0264345

**Published:** 2022-02-23

**Authors:** Yang Lv, Min Wu, Roger C. Shouse

**Affiliations:** 1 College of Teachers, Chengdu University, Chengdu, China; 2 School of Public Administration, Sichuan University, Chengdu, China; 3 Department of Education Policy Studies, Pennsylvania State University, University Park, PA, United States of America; Rzeszow University of Technology: Politechnika Rzeszowska im Ignacego Lukasiewicza, POLAND

## Abstract

At a time when vocational education is seen as critical for national development, concern grows regarding how weak organizational culture and occupational commitment may threaten the production of quality graduates and teachers within the field. The failure of vocational institutions to effectively create human capital likely threatens the connections between Chinese industry and its educational institutions. This study thus explores how these connections are influenced by organizational and occupational factors. A multi-layer linear model is employed on data collected from 406 teachers from 69 Chinese vocational colleges and universities. Results suggest that organizational cultural positively influences industry-university cooperative behavior through the construction of strong occupational commitment and job involvement. This study not only enriches and expands new knowledge and academic perspectives, but also provides feasible policy suggestions to help guide educational administrators toward the improvement of vocational education.

## 1. Introduction

The systematic balancing and coordination of different types of education is a critical concern for Chinese educational reform. It is particularly salient with respect to the special goals and functions of vocational education and its links to Chinese industry [1]. Although the Belt and Road Initiative (BRI) is an important force linking industries, universities, and research institutions, its connections to vocational education remain unclear. At the same time, the February 13, 2019 declaration holds that education reform cannot take place without the modernization of vocational education. It is thus argued that a primary path toward this can be found in the effective integration of human capital, organizational structure, and production [2, 3].

In other words, high-quality workers and technical skills are cultivated in the process of school-enterprise interaction [4], one key component of which is the supply of highly skilled and engaged teachers [5]. Yet while it seems especially important to study factors that support this supply, studies on vocational school-industry cooperation mainly focus on structural factors such as policy orientation, standards, and management [6–8]. In contrast, studies related to social-psychological factors such as cognition, emotion, and behavior are still lacking. Though many scholars’ studies reveal the influences of individual factors like teachers’ gender, age, title, and seniority on industrial participation [9–15], few have focused on how interaction between individual and organizational characteristics help generate the forces necessary for dynamic learning-industry connections to thrive [16–19].

Supportive of this idea, researchers such as Thomas and Velthouse [20] highlight the attitudes and meanings employees attach to their work, which in turn serve as intrinsic factors promoting motivation, job attachment, satisfaction, creativity and skill [21–23]. In addition, meanings are constructed and attitudes emerge within the situational context in which work occurs [24]. A proper balance and integration of organizational structure, context, and individual factors are thus said to contribute to job satisfaction [25], career happiness [25], teaching performance [26] and career retention and stability [27]. Such factors, it is further suggested, may have a positive impact on cooperative behavior [28]. Typically conducted outside of China, however, such studies may fail to consider difference in historical and cultural traditions, management styles and organizational environments of Chinese vocational education [29, 30].

China’s need for vocational education development is nonetheless clear. A need exists for high quality teachers who can creatively integrate production, learning, and research in ways that not only apply theory to practice, but also encourage the development of new theories supporting continuous improvement in both teaching and industrial practice [2].

Clearly, research is needed to examine the underpinnings of industry-academic integration within the unique context of Chinese social, organizational, and management norms. This study thus examines and highlights how structural factors (such as organizational culture) in higher vocational educational institutions interact with individual teacher characteristics (such as occupational commitment) to influence the connections between China’s industries and the vocational teachers upon which they depend. Such skills, and the workers they help produce, are in part generated by what may be called school-enterprise, learning-industry, or academic-industry interaction.

## 2. Theoretical framework and research hypothesis deduction

### 2.1 Occupational commitment and industry-university cooperation

Occupational commitment, or career commitment, is understood as the relative strength of one’s sense of identification with a line of work, as well as one’s desire and obligation to remain in his or her career or organization [31]. Other researchers that commitment is also a function of one’s unwillingness to change careers due to economic or emotional dependence, as well as one’s internalization of an internalization of the profession’s social norms [32]. Dik and Duffy [33] emphasize the importance of commitment to work while, in the field of education, Weifang and Yi [34] point out its link to teachers’ engagement, desire to remain in their career, willingness to improve professional skills, and to contribute to the institutional advancement of teaching, learning, and their social application.

In the area of vocational education, such contributions are reflected in industry-academic cooperation, the degree to which teachers remain current and creative in their knowledge of industrial trends, techniques, and opportunities. In light of China’s educational, economic, and social needs and goals, occupational commitment may be most critical in the area of vocational education. We are thus led to the following hypothesis:

**H1**: Occupational commitment of teachers in higher vocational education has a significant positive impact on their industry-academic cooperation behaviors.

### 2.2 Organizational cultural atmosphere and industry-university cooperation

Various studies suggest the sort of organizational cultural characteristics likely to promote professional commitment, job satisfaction and, in turn, industry-academic cooperation. Tartari et al. [35], for example, establish the point that good organizational atmosphere promotes expertise and research in the development of industrial fields. In related fashion, Ayyildiz and Yilmaz [36] report that organizations motivate members toward innovative behavior by encouraging new ideas and free speech. Costa et al. [36] argue that clan culture is more important in the organization. Huang et al. [37] further reports that such encouragement, coupled with resources and supportive management practices are the most important predictors of employee creative performance.

Similar studies indicate Shalley and Gilson [38] point out that employees are more likely to demonstrate creative work and innovation when employers express value for such work and provide adequate resources. In the academic realm, colleges and universities possess the capability to promote innovative values, rules, and norms among their members [39–41]. The sum of such characteristics can be said to form a culture of diverse thinking among diverse employees which in turn promotes faculty satisfaction, organizational identification, and improved performance [42, 43]. Such studies give rise to the following hypothesis:

**H2**: The organizational culture atmosphere of higher vocational institutions has a significant positive prediction effect on teachers’ industry-learning cooperation behavior.

### 2.3 The mediating effect of job involvement

In addition to organizational culture, individual cognitive characteristics, such as occupational commitment and involvement, are said to positively influence the quality of professional activity. Ryan and Deci’s [44] organic integration theory suggests this occurs as organization members internalize recognized norms, values, rules, and other external motivators. In vocational higher education settings, such internalization is expected to assume responsibility for high quality performance, hold positive emotional attachment to their work, and form more active job involvement. Studies reveal a positive connection among job involvement, engagement, emotional attachment, career commitment, and positive work ethic [45–47]. Findings by Richter et al. [48] support these connections within educational settings. As teachers internalize the conditions of their work, satisfaction, commitment, and professional involvement are likely to increase. We are thus led to the following hypothesis:

**H3**: vocational commitment of teachers in higher vocational colleges has a significant positive predictive effect on their job involvement.

### 2.4 Job involvement and improved work performance

A key purpose of studying teachers’ job involvement is to understand how it shapes the quality of worker performance generally [49, 50], and particularly in the field of higher education [22, 29, 51–53]. This connection, pose questions. Schaufeli et al. [54] suggest, the connection between involvement and performance likely emerges from a spiral of mutual influence and interaction. Another issue involves the fact that involvement itself is used as a measure of performance. It is thus important to clarify the specific types of performance that involvement is likely to encourage or improve.

In higher education, job is typically judged in terms of teaching and research, but also in terms of practical contributions to a larger community. This type of “practice performance” has become especially important in vocational education institutions, mainly in reference to industry-learning cooperative activity, sometimes referred to as academic extension or outreach. We thus infer that vocational college teachers’ increased involvement in such activity will likely lead to its expansion and improvement. Accordingly, the following research hypothesis is proposed:

**H4**: The job involvement of teachers in higher vocational colleges has a significant positive predictive effect on their industry-school cooperation behavior.

### 2.5 Indirect effects of culture and involvement

In addition to their direct effects on academic extension (i.e., industry-learning cooperative behavior), organizational culture and job involvement have been identified as exerting interactive impact on the processes through which these effects occur [55]. For example, whether a “supportive” organizational culture encourages academic extension likely depends on its normative and structural inclinations and dispositions. An organization in which employee job “commitment” or “involvement” are understood to be primarily reflected in the attainment of grants and publications may be less likely to promote creative academic outreach than one that supports risk-taking and cognitive diversity [56]. Similarly, an organization driven largely by competition and individuality may be less likely to promote creativity or risk taking than one with an atmosphere of collectivism and communality [57]. We are thus led to the following hypotheses:

**H5**: The job involvement of teachers in higher vocational institutions has an intermediary effect between their occupational commitment and industry-academy cooperation behavior.

**H6a**: The organizational cultural atmosphere in higher vocational institutions has a significant moderating effect on the relationship between teachers’ occupational commitment and industry-academy cooperation behavior.

**H6b**: The organizational cultural atmosphere in higher vocational institutions has a significant moderating effect on the relationship between teachers’ job involvement and industry- academy cooperation.

## 3. Research methods

Hierarchical Linear Modeling (HLM) is employed in this study to examine the influencing factors of organizational and individual occupational factors on industry—academy cooperation. HLM is a statistical method appropriate for analyzing how the variation of a dependent variable is influenced by nested characteristics (e.g., individuals within groups). Traditional regression analysis, which places individual and group variables into a single model, cannot adequately estimate the influence of nested characteristics upon a dependent variable [58]. HLM examines these effects separately within a single equation. This study uses HLM6.08 software for statistical analysis.

### 3.1 Sample and data collection

Teachers in higher vocational institutions served as the objects of investigation. A total of 69 higher vocational institutions were investigated across 13 provinces, municipalities directly under the central government, and 22 cities. The research was divided into two stages. The first of these involved a pilot study of 54 vocational teachers from 10 vocational institutions. This pilot study led to a second stage in which the questionnaires were revised and distributed to a larger sample of teachers. In this survey, 456 questionnaires were collected, of which 406 were valid. Verbal consent was taken from the respondent before every interview.

In this study, non-repeated sampling is used to determine the sampling error, and the calculation formula is as follows:

$$\mu_{x}=\frac{\sigma_{x}}{\sqrt{n}}\sqrt{1-\frac{n}{N}},\;\sigma_{x}=\sqrt{\frac{\sum {\left(x-\bar{x}\right)}^{2}f}{\sum f}}$$

The mean sampling error of this study is 0.135, which indicates that the sample is highly representative. The basic information about demographic characteristics is shown in Table 1.

**Table 1:** Descriptive statistics of sample demographic characteristics.

Variable	Range	Frequency	Percentage
Sex	Male = 1	234	57.6
	Female = 0	172	42.4
Title	Teaching assistant = 1	155	38.2
	Lecturer = 2	161	39.7
	Associate Professor = 3	77	19.0
	Professor = 4	13	3.2
Teaching age	Within 5 years = 1	200	49.3
	5–10 years = 2	70	17.2
	10–15 years = 3	71	17.5
	More than 15 years = 4	65	16.0
Administrative duties	Yes = 1	120	29.6
	No = 0	286	70.4

### 3.2. Definition and measurement of variables

#### 3.2.1 Dependent variable

The dependent variable in this paper is industry-academy cooperation, which refers to 11 types of industrial activities summarized by in previous research [59, 60]. A participation index of these activities was construct based on participation frequency and a difficulty coefficient. Previous studies indicate that these selected types of industrial activities fall into two categories; organization-related and individual-related. For purposes of this study, seven types of industrial activities comprised our index. The index was scored on a 5-point Likert scale, with scores from 1 to 5 representing “strongly disagree”, “disagree”, “generally”, “agree” and “completely agree”.

#### 3.2.2 Independent variables

*3*.*2*.*2*.*1 Occupational commitment*. The key independent variable in this paper is occupational commitment. Professional commitment refers an organization member’s positive willingness to take on and fulfill professional roles and duties, not only in terms of state of mind, but also as expressed in external behavior performance. In this study, teacher’s occupational commitment is represented by their expressed willingness and reasons for engaging in teaching and research. Under the guidance of the theoretical model, different scholars have compiled different measurement tools which have been verified in many studies with good reliability and validity. This paper adopts the five-item Likert scale for occupational commitment [61]. The assessment was scored on a 5-point Likert scale that ranges from 1 to 5 representing “strongly disagree”, “disagree”, “generally”, “agree” and “completely agree”.

*3*.*2*.*2*.*2 Job involvement*. This study adopts the Utrecht Work Engagement Scale (UWES) to measure job involvement [54]. The scale consists of three subscales (vitality, dedication, and focus) with items measured on a 5-point Likert scale ranging from 1 to 5. The higher the score, the higher the teacher’s job involvement.

*3*.*2*.*2*.*3 Organizational culture*. This study conceives of organizational culture as the degree to which management encourages an atmosphere that is open, harmonious, and supportive of creative approaches to teaching and improved work performance. Though scholars have developed different scales to measure organizational culture, this study adopts and integrates indicators developed by Guerrero and Urbano [62] and Scott and Bruce [39] to measure overall atmosphere, resource support, and teamwork. The scale was scored on a 5-point Likert scale that ranges from 1 to 5 representing “strongly disagree”, “disagree”, “generally”, “agree” and “completely agree”.

#### 3.2.3 Control variables

In accord with previous studies indicating a correlation between individual and organizational characteristics, this study includes teacher-level controls for gender, professional title, teaching age and administrative position.

### 3.3 Reliability and validity analysis

SPSS version 22 was used to test the reliability and validity of the incorporated scales. As shown in Table 2, Cronbach’s coefficient of variation is 0.91, 0.92, 0.90 and 0.87 respectively, indicating that the scale has good reliability. Before the factor analysis, the KMO test and Bartlett’s spherical test were first performed. The KMO value was 0.91 (p < 0.001), indicating that the results were suitable for factor analysis. Principal component analysis was then used to extract and explore initial factors. Maximum variance method was used to reasonably explain the factor groups. After rotation, the measurement items with factor loads greater than 0.5 were retained. Next, the component reliability and convergent validity of the sample data measurement were further checked. The component reliability (CR) of all variables was higher than 0.70, indicating that the sample data measurement had good component reliability. In addition, the estimated values of standardized factor loading (standard deviation) points of the observation variables used for measuring items and the corresponding variables were all above 0.5, squared multiple correlation (SMC) values were all greater than 0.3, and average variance extracted (AVE) values were all greater than 0.5, indicating that the sample data measurement had good convergent validity.

**Table 2:** Results of reliability and validity.

Variable	Measuring item	Std. dev	Alpha	SMC	CR	AVE
Occupational commitment						
It means a lot to me to stay in the field of vocational education.		0.85	0.91	0.72	0.91	0.71
I must remain faithful to the work of vocational education.		0.82		0.67		
If I left the vocational education work now, I would feel very empty.		0.87		0.74		
After receiving vocational education, teachers should not change careers at will.		0.85		0.71		
Job involvement						
At work, I feel like I’m bursting with energy.		0.83	0.92	0.69	0.92	0.66
I think my work is purposeful and meaningful.		0.82		0.68		
At work, even when things don’t go well, I always persevere.		0.83		0.68		
I can work long hours at a time.		0.78		0.61		
When I work, I forget myself.		0.78		0.61		
I will immerse myself in my work.		0.82		0.68		
Organizational cultural atmosphere						
Our school will encourage the support and cooperation of the link social service agencies.		0.84	0.89	0.71	0.92	0.70
Teachers in our school are encouraged to be creative and have ideas.		0.84		0.71		
Teachers in our school exchange ideas freely and openly with each other.		0.79		0.62		
The leaders of our school attach great importance to the contribution of every teacher and the transformation of academic achievements.		0.84		0.71		
Our school leaders pay great importance to the research with applied value.		0.85		0.73		
Industry-learning cooperation behavior						
I will try to participate in joint or collaborative research (funded by the enterprise).		0.80	0.87	0.63	0.88	0.60
I will try to apply for projects jointly with industry to study government-funded projects.		0.82		0.67		
I will try to take students to enterprises for internship or part-time training.		0.76		0.58		
I attend conferences and forums organized by the industry.		0.80		0.64		
I will try to provide technical advice or technical services to the industry.		0.69		0.48		

Note: SMC = Squared Multiple Correlation; CR = Component Reliability; AVE = Average Variance Extracted.

As shown in Table 3, the AVE square root of each variable is greater than the correlation coefficient between the variable and other variables, indicating that the sample data measurement has good discrimination validity.

**Table 3:** Results of variable discrimination validity.

Variables	AVE	OC	JI	OCA	IICB
Occupational commitment (OC)	0.71	1			
Job involvement (JI)	0.66	0.47	1		
Organizational cultural atmosphere (OCA)	0.70	0.15	0.06	1	
Industry-learning cooperation behavior (IICB)	0.60	0.45	0.44	0.35	1

### 3.4 Descriptive statistics of variables

The mean, standard deviation and correlation coefficient matrix of the variables are shown in Table 4.

**Table 4:** Mean value, standard deviation and correlation coefficient of variables.

Variable	Mean	Std dev.	OC	JI	OCA	IICB
Occupational commitment (OC)	3.43	1.09	1			
Job involvement (JI)	3.5	0.1	0.42**	1		
Organizational cultural atmosphere (OCA)	3.36	0.83	0.14**	0.06	1	
Industry-learning cooperation behavior (IICB)	3.58	0.9	0.39**	0.39**	0.31**	1

Note

**p≤0.01. Source: Field survey.

### 3.5 Model construction

Multi-level modeling is an analytic strategy that begins with the construction of a “level-1” model that gages the effects of, in this case, individual-level variables. The intercept of the resulting equation is then modeled in a “level-2” (group-level) equation gaging the effects of, here, organizational-level variables.

A “null model” was first constructed to determine the extent of group-level variation (see [63]). HLM6.08 was used for model validation. Based on our hypotheses, further models were then constructed. In the models presented below, y = COOP; y_ij_ is the level of cooperative behavior for individual “i” in school “j”. β_0j_ represents the level of cooperative behavior in school “j”. r_ij_ represents the unexplained variation for individual “i” in school “j”.

Null Model 0–1: Level 1: y_ij_ = β_0j_ + r_ij_

                        Level 2: β_0j_ = γ_00_ + μ_0j_; where γ_00_ represents the average level of cooperative behavior across sampled schools and μ_0j_ the unexplained level-2 variance. Next, we model the individual variation of both job involvement and cooperative behavior.

                        Null Model 0–2: Level 1: INVOLV_ij_ = β_0j_ + r_ij_

                        Level 2: β_0j_ = γ_00_+μ_0j_

Model 1–1: Level 1: COOP_ij_ = β_0j_ + β_1j_ (SEX_ij_)+ β_2j_ (RANK_ij_) + β_3j_(EXPER_ij_)+β_4j_(ADMIN_ij_) +β_5j_(committ_ij_) + r_ij_

                    Level 2: β_0j_ = γ_00_+μ_0j_

                            β_1j_ = γ_10_

Model 1–2: Level 1: COOP_ij_ = β_0j_ + β_1j_(SEX_ij_) + β_2j_(RANK_ij_) + β_3j_(EXPER_ij_)+β_4j_ (ADMIN_ij_)+β_5j_(COMMIT_ij_) + β_6j_(INVOLV_ij_) + r_ij_

                    Level 2: β_0j_ = γ_00_+μ_0j_

                            β_1j_ = γ_10_

Model 1–3: Level 1: INVOLV_ij_ = β_0j_ + β_1j_(SEX_ij_) + β_2j_(RANK_ij_) + β_3j_(EXPER_ij_)+β_4j_(ADMIN_ij_) + β_5j_(COMMIT_ij_) + r_ij_

                    Level 2: β_0j_ = γ_00_+μ_0j_

                            β_1j_ = γ_10_

We then constructed level-2 models for cooperative behavior. Model 3 adds level-1 terms representing the interaction between RANK and ADMIN.

Model 2: Level 1 COOP_ij_ = β_0j_ + β_1j_(SEX_ij_) + β_2j_(RANK_ij_) + β_3j_(EXPER_ij_)+β_4j_(ADMIN_ij_) + r_ij_

                    Level 2: β_0j_ = γ_00_+γ_01_(ORGCUL_j_)+μ_0j_

Model 3: Level 1COOP_ij_ = β_0j_ + β_1j_(SEX_ij_) + β_2j_(RANK_ij_) + β_3j_(EXPER_ij_) + β_4j_(ADMIN_ij_)) + β_5j_(COMMIT_j_)+r_ij_

                    Level 2: β_0j_ = γ_00_+γ_01_(ORGCUL_j_)+μ_0j_

                            β_5j_ = γ_50_+γ_51_(ORGCUL_j_ -$\bar{ORGCUL}$.)+μ_5j_

Model 4: Level 1: COOP_ij_ = β_0j_ + β_1j_(SEX_ij_) + β_2j_(RANK_ij_) + β_3j_(T_ij_) + β_5j_(INVOLV_ij_ -${\bar{INVOLV}}_{.j}$) + r_ij_

                    Level 2: β_0j_ = γ_00_+γ_01_(ORGCUL_j_)+μ_0j_

                            β_5j_ = γ_50_+γ_51_(ORGCUL_j_-$\bar{ORGCUL}$.)+μ_5j_

### 3.6 Ethical approval

This study followed all ethical guidelines and standards of the research. Ethical approval was obtained from the ethics review committee of Sichuan University, Chengdu, China. Besides, verbal approval was obtained from each interviewee before an interview. This study also maintained the confidentiality of the interviewee.

## 4. Results of model analysis

According to the research hypothesis, the analysis results were divided into main effect, mediating effect, and regulating effect results. Standard errors are inside the parentheses. The results of model operation are shown in Tables 5–7.

**Table 5:** Main effect test results.

Model variables	Industry-learning cooperation behavior				Job involvement	
	0–1	1–1	1–2	2	0–2	1–3
Intercept	3.59(0.08)***	2.43(0.17)***	1.99 (0.19)***	2.01(0.47)***	3.49(0.08)***	2.21(0.17)***
Individual level						
Sex		-0.37(0.06)***	-0.30(0.06)***	-0.35(0.07)***		-0.36(0.07)***
Title		0.03(0.05)	0.03(0.05)	0.03(0.05)		0.01(0.05)
Teaching age		0.12(0.03)***	0.09(0.03)**	0.11(0.03)***		0.15(0.03)***
Administrative duties		-0.07(0.09)	-0.07(0.09)	-0.07(0.09)		-0.04(0.08)
Occupational commitment		0.30(0.04)***	0.24(0.04)***			0.33(0.04)***
Job involvement			0.19(0.04)***			
Organizational level						
Organizational cultural atmosphere				0.44(0.14)**		
Statistic						
R^2^	0.47	0.37	0.36	0.43	0.62	0.53

Note

***p<0.001

**p≤0.01, *p≤0.05. Source: Field survey.

**Table 6:** Results of mediation effect test.

Model variables	Industry-academy cooperation (1–1)	1–3	1–2
Intercept	2.43(0.17)***	2.21(0.17)***	1.99(0.19)***
Sex	-0.37(0.06)***	-0.36(0.07)***	-0.30(0.06)***
Title	0.03(0.05)	0.01(0.05)	0.03(0.05)
Teaching age	0.12(0.03)***	0.15(0.03)***	0.09(0.03)**
Administrative duties	-0.07(0.09)	-0.04(0.09)	-0.07(0.09)
Occupational commitment	0.30(0.04)***	0.33(0.04)***	0.24(0.04)***
Job involvement			0.19(0.05)***
R^2^	0.37	0.53	0.36

Note

***p<0.001

**p≤0.01, *p≤0.05. Source: Field survey.

**Table 7:** Effects on cooperative behavior.

Model variables	0–1	2	3	4
Intercept	3.59(0.08)***	2.01(0.47)***	3.46(0.12)***	3.54(0.12)***
Individual level				
Sex		-0.35(0.07)***	-0.37(0.07)***	-0.24(0.06)***
Title		0.03(0.05)	0.03(0.05)	0.02(0.05)
Teaching age		0.11(0.03)***	0.13(0.03)***	0.06(0.03)*
Administrative duties		-0.07(0.09)	-0.08(0.09)	-0.06(0.09)
Occupational commitment			0.31(0.04)***	
Job involvement				0.28(0.04)***
Organizational level				
Organizational cultural atmosphere		0.44(0.14)**	0.43(0.14)**	0.44(0.14)**
Organizational culture × occupational commitment			0.19(0.09)*	
Organizational culture × job involvement				0.20(0.09)*
Statistic				
R^2^	0.470	0.426	0.367	0.378

Note

***p<0.001

**p≤0.01

*p≤0.05. Source: Field survey.

### 4.1 Main effects

Table 5 presents the results of two separate regression analyses. Its first four columns examine effects on cooperative behavior; the last two columns on job involvement. With respect to cooperative behavior, columns two and three reveal the effects of individual-level controls, occupational commitment, and job involvement. In case of Industry-learning cooperation behavior, 0–1, 1–1, 1–2 and 2 model means industry-learning cooperation behavior_ij_ = β_0j_+r_ij_, industry-learning cooperation behavior_ij_ = β_0j_+β_1j_(Sex_ij_)+ β_2j_(Title_ij_) + β_3j_(Teaching age_ij_)+β_4j_(Administrative duties_ij_)+β_5j_(occupational commitment_ij_) +r_ij_, industry-learning cooperation behavior_ij_ = β_0j_+β_1j_(Sex_ij_)+β_2j_(Title_ij_)+β_3j_(Teaching age_ij_)+β_4j_(Administrative duties_ij_)++β_5j_(occupational commitment_ij_) +β_6j_(Job involvement_ij_)+r_ij_ and industry-learning cooperation behavior_ij_ = β_0j_+β_1j_(Sex_ij_)+β_2j_(Title_ij_)+β_3j_(Teaching age_ij_)+β_4j_(Administrative duties_ij_)+r_ij_ respectively. In case of job involvement, 0–2 and 1–3 indicate Job involvement_ij_ = β_0j_+r_ij,_ and Job involvement_ij_ = β_0j_+β_1j_(Sex_ij_)+β_2j_(Title_ij_)+β_3j_(Teaching age_ij_)+β_4j_(Administrative duties_ij_)++β_5j_(occupational commitment_ij_) +r_ij_ respectively. Both commitment and involvement were found to be significantly related to cooperative behavior (0.24 and 0.19 respectively). Column four indicates the positive effect of organizational culture on cooperative behavior (0.44).

The final two columns of Table 6 reveal how individual characteristics relate to job involvement. Based on model 0–1, control variables and independent variables at the organizational level were added in model 2. The results showed that organizational cultural atmosphere had a significant impact on industry-academy cooperation behavior (0.44, P<0.01). Model 1–3 added the independent variable occupational commitment on the basis of model 0–2, and the results showed that occupational commitment had a significant impact on job involvement (= 0.33, P<0.001), and confirmed the research hypothesis H3.

In the case of H1, the value 5 shows that occupational commitment significantly impacts industry-learning cooperation behavior (0.30, P < 0.001). It suggests that the higher the degree of occupational identification, the stronger the occupational emotional dependence. It means the better the performance of occupational commitment, the stronger the willingness to participate in industry-learning cooperation behavior (Table 5).

In the case of H2, the analysis shows that the organizational cultural environment significantly impacts industry-learning cooperation behavior (0.44, P < 0.01). It suggests that the organizational culture environment influences employees’ behaviors, such as organizational climate, organizational culture, structure, leadership style, man-machine relationship, organizational system, etc., which influence individuals’ choice of industry-learning cooperation behavior to a certain extent.

In the case of H4, the analysis shows that work involvement significantly impacts industry-learning cooperation behavior (0.19, P < 0.001). It suggests that the more individuals put their energy into work, and actively demonstrate their self-worth. It indicates that the better the performance of work involvement, the greater the impact of individual choice of industry-learning cooperation behavior.

### 4.2 Mediation effects

In Table 6, Model 1–3 and 1–2 indicate Job involvement_ij_ = β_0j_+β_1j_(Sex_ij_)+β_2j_(Title_ij_)+β_3j_(Teaching age_ij_)+β_4j_(Administrative duties_ij_)++β_5j_(occupational commitment_ij_) +r_ij_ and industry-learning cooperation behavior_ij_ = β_0j_+β_1j_(Sex_ij_)+β_2j_(Title_ij_)+β_3j_(Teaching age_ij_)+β_4j_(Administrative duties_ij_)++β_5j_(occupational commitment_ij_) +β_6j_(Job involvement_ij_)+r_ij_ respectively. The values of model 1–1 showed that occupational commitment had a significant impact on industry-academy cooperation behavior (beta = 0.304, P < 0.001) (Table 6). The results of model 1–3 showed that occupational commitment had a significant impact on job involvement (beta = 0.325, P < 0.001).

Model is 1 to 2 in 1–1 joined the intermediary variable job involvement, and the results showed that job involvement had a significant effect on co-operative behavior (beta = 0.192, P < 0.001). It indicated a mediation effect exists in the professional commitment to the effects of the co-operation behavior and confirmed research hypothesis H5.

### 4.3 Regulating effects on cooperative behavior

Model 3 reveals the significant interactive effect of organizational culture and occupational commitment (Table 7). The result showed that organizational culture atmosphere between occupational commitment and co-operative behavior had a positive regulatory role (beta = 0.186, P < 0.05) that reflected the organizational culture atmosphere.

**Fig 1:**
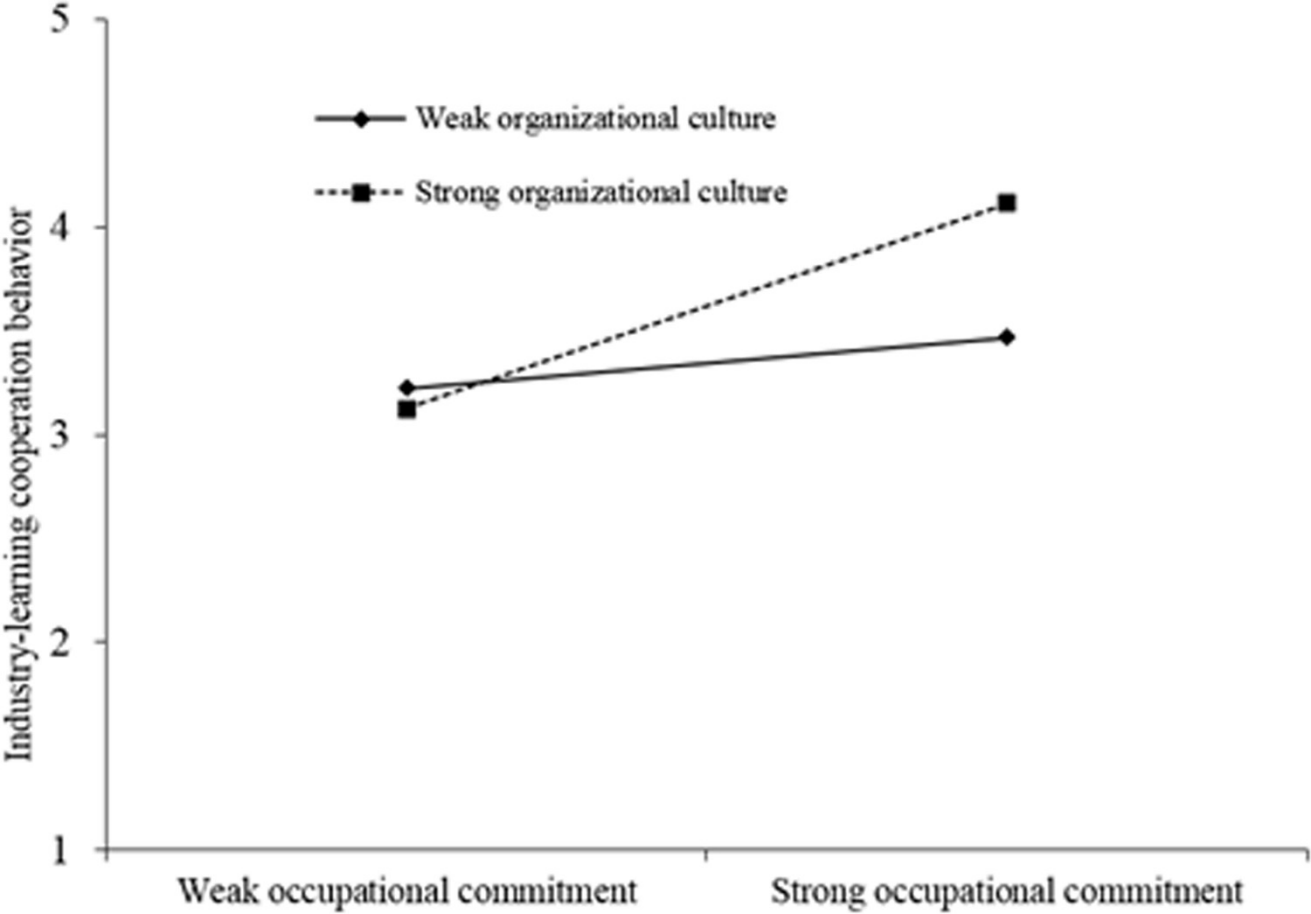
Cross-layer adjustment diagram of organizational cultural atmosphere and occupational commitment.

The better commitment ensures to the greater influence of co-operation behavior. That confirmed research hypothesis H6a. The specific effect diagram is shown in Fig 1.

**Fig 2:**
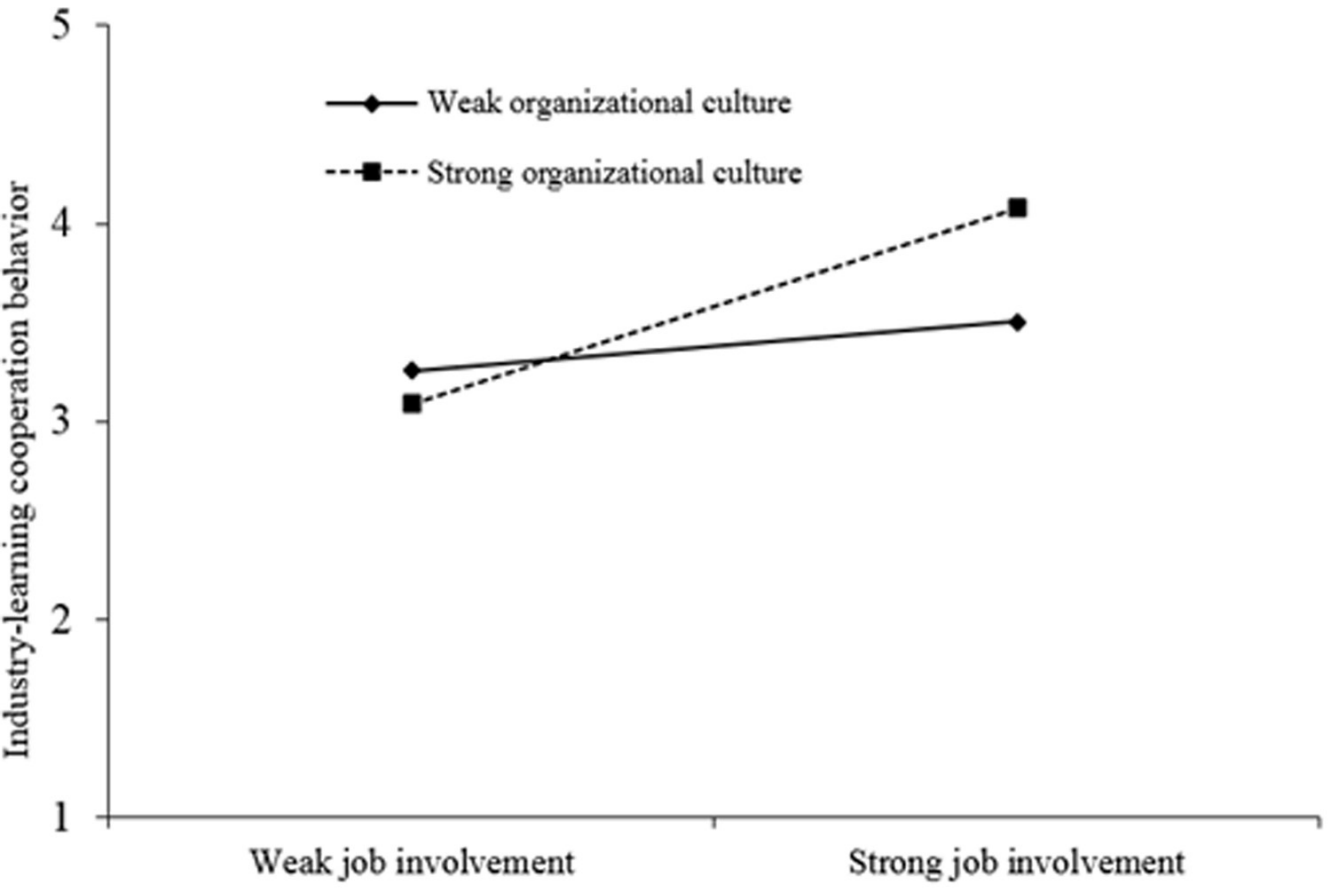
Cross-layer adjustment diagram of organizational cultural atmosphere and job involvement.

Model 4 focused the regulating variable organization culture × job involvement. The values indicated that organizational culture atmosphere between job involvement and co-operation behavior had a positive regulatory role (beta = 0.200, P < 0.05). It reflected the organizational culture atmosphere like the better the work can ensure the greater impact on co-operation behavior and confirmed research hypothesis H6b. The specific effect diagram is shown in Fig 2.

## 5. Discussion and conclusion

Aiming at the research on industry-learning cooperation behavior of teachers in higher vocational colleges, this study turns into shift attention and the perspective to the career development of teachers in higher vocational colleges. The question of how organizational culture, other organizational variables, and individual characteristics influence the academic outreach of faculty members has not been well-addressed in prior literature. This question, however, is of key importance for leaders and policy makers seeking to vitalize and revitalize the links between vocational education and a nation’s industrial innovation and effectiveness. Our study contributes to overall understanding of this issue by examining this influence from a multi-level perspective.

Because these problems cannot be said to have been understood clearly in theory or even in practice, they are difficult to be solved systematically and efficiently. Therefore, co-operative behavior influence factor for higher vocational teachers research is not only to analyze from the macro level, but also from the micro level is analyzed, existing however, involved in macro level variables analysis is not the real from the perspective of statistical evidence as a high level of variables, mostly adopt the method of single level carries on the statistical test, this would violate regression analysis on the assumption of independent variables. With the progress of data analysis technology, the structure of cross-layer inspection at different levels and between different levels has gradually gained researchers’ attention. In addition, the existing co-operative behavior of higher vocational teachers research around the influential factors of teachers’ gender, job title, teaching age, qualifications and other demographic variables such as factor analysis on the level of objective [64], few focus on higher vocational teachers’ cognition, emotion, behavior, such as teachers’ own quality professional commitment and job involvement is analyzed.

A further contribution of this study lies in its emphasis on how vocational academic-industry connections and extension may hold more promise for China’s economic improvement than simple reliance on academic application.

However, at present, the academic environment in China is more emphasis on academic application, the external neglect and the depreciation of academic peers lead to the phenomenon that teachers in higher vocational colleges are not active in industrial participation and cooperation and are passive in work. Therefore, based on the first-hand data collected by the research group, this study comprehensively adopts a variety of empirical analysis methods to explore the occupational commitment and work commitment of teachers at the individual level, the organizational culture atmosphere of higher vocational colleges at the organizational level, and the internal mechanism of cross-level influence on the industry-learning cooperation behavior of higher vocational teachers.

### 5.1 Key insights of the study

At the individual level, the results of this study show that the higher the level of teachers’ occupational commitment and job involvement, the better their industry-school cooperation behavior performance. The research result Torrance [65] and other scholars indicating that the degree to which a teacher is willing to change his or her profession has an effect on his or her cooperative behavior, which is partly reflected by his or her job involvement. This further reflects the importance of promoting teachers’ occupational commitment [65]. Job involvement and commitment promote willingness to change their profession, thus promoting cooperative behavior. This supports the view of traditional psychological theories that motivated behavior comes from the satisfaction and pleasure gained by individuals engaging in valuable activities.

At the organizational level, the results of this study show that the better the organizational cultural atmosphere of vocational colleges, the more positive is teachers’ industry-learning cooperation behavior. This result echoes the findings of Péter-Szarka [66], and Zhou et al. [67] that the climate of the school organization and the availability of resources affect the performance of teachers. This suggests that when management provides adequate resources, promotes harmony, and supports teacher sharing and innovation, teacher work performance is stimulated in positive ways such as professional creativity and outreach. Our results also suggest that such a nurturing culture and climate in vocational colleges promotes teachers’ occupational commitment and job involvement, which in turn promote improved industry-school cooperation behavior. Harmonious and supportive organizational culture encourages enhanced teacher commitment, emotional investment, involvement, and productive cooperation behavior. This further highlights the importance of the organizational culture atmosphere.

### 5.2 Theoretical and practical significance

#### 5.2.1 Theoretical significance

The traditional single-level regression models typically used in the field of higher vocational education research are inadequate in revealing the multilevel effects of organizational culture. Ours is one of the few studies that considers the nested impact of individual and organizational variables and analyzes them through a hierarchical model. This study will thus be helpful to future research and understanding in the field of higher vocational education. Our study contributes to theoretical understanding by supporting the idea that individual behavior is not only influenced by external objective factors, but also by internal individual factors and their interaction with organizational context.

#### 5.2.2 Practical significance

The practical implications of this study for higher vocational education are also of great value. Based on the above research findings, the promotion of industry-learning cooperation among teachers in higher vocational colleges is realized through the stimulation of teachers’ professional interest and occupational commitment. By implication, the effectiveness of higher vocational education is likely improved, made more authentic, and more socially relevant through changes in organizational culture and climate. We are confident that this will help strengthen vocational commitment of teachers, rebuild professional confidence, promote professional satisfaction, and increase active engagement in industry-school cooperation.

In addition, our findings support a synthesis of theory and practice as well as a shift toward more participatory practical training. These should provide a supportive platform for professional initiative, creativity, as well as a new template for the preparation of higher vocational teachers. It thus seems urgent to incorporate such change into the study and application of management theory. The cultural pursuit of harmony, openness and fairness in colleges and universities is found to be a promising institutional design strategy. It promises to encourage higher vocational teachers to innovate and extend their practice, thus promoting the overall transformation of higher vocational academic scholarship and achievement. In this way, higher vocational teachers can make the best use of their talents, including in the form of high quality industry-learning cooperation.

#### 5.2.3 Research prospects

In terms of research content, we believe that in addition to organizational cultural atmosphere, there are other salient macro or meso-level factors for future studies to explore. These include studies of professional motivation and behavior, personality, and experience. Equally if not more important would be studies of the processes through which academic or other institutions may shift their cultures toward the more humanistic forms described in this paper. Future research can also enrich and improve research designs, thereby contributing more comprehensively to academic achievement and scholarship in the field of higher vocational education.

## Supporting information

S1 Questionnaire (DOCX)

S2 Questionnaire (DOCX)
