# Supplementary material for: Impact of organizational culture, occupational commitment and industry-academy cooperation on vocational education in China: Cross-sectional Hierarchical Linear Modeling analysis
Source: PLoS One. 2022 Feb 23;17(2):e0264345. doi: 10.1371/journal.pone.0264345 (PMC8865651; doi:10.1371/journal.pone.0264345)
Supplement: S1 Questionnaire — (DOCX) [file pone.0264345.s001.docx]

**职业院校教师产业参与行为调查问卷**

您好！这是一份由四川大学公共管理学院研究团队进行的问卷调查，目的在于了解职业院校教师学者产业参与行为的影响因素。请您根据实际情况在适当的选项中打“√”。本问卷填答约需借用您5-10分钟宝贵时间，我们非常珍惜和重视您的意见，您所回答的一切资料仅供研究之用，本研究将遵守《中华人民共和国统计法》第十三章第十四条的规定，对您回答的所有信息保密，请你不用顾虑，放心作答。万分感谢您的协助与合作！

以下描述，您请在符合情况的选项上打“√”：

一、基本信息

1.您的性别？

（1）男

（2）女

2.您的职称？

（1）助教

（2）讲师

（3）副教授

（4）教授

3.您的教龄？

（1）5年以下

（2）5-10年

（3）10-15年

（4）15年以上

4.您是否担任行政职务？

（1）是

（2）否

5.您所在学校的城市和名称？（为了方便分组，劳烦各位老师务必填写，万分感激！）

以下描述，请您根据问题的提示语回答自己的想法。数字1至5分别代表队问题内容的完全不同意、不同意、一般、同意、完全同意。请根据回答内容，在您认为最能代表您真实想法的分数选项下打“√”：

二、调查内容

1.我留在职业教育领域学界对我而言是意义重大的。

完全不同意|_1_|_2_|_3_|_4_|_5_|完全同意

2.我必须对职业教育工作保持忠诚。

完全不同意|_1_|_2_|_3_|_4_|_5_|完全同意

3.如果现在离开职业教育工作，会让我感到很空虚。

完全不同意|_1_|_2_|_3_|_4_|_5_|完全同意

4.教师接受职业教育培养后，不应该随意转业。

完全不同意|_1_|_2_|_3_|_4_|_5_|_完全同意

5.职业教育工作对我的个人形象塑造助益良多。

完全不同意|_1_|_2_|_3_|_4_|_5_|完全同意

6.我喜欢接受和应对职业教育工作中难题的挑战。

完全不同意|_1_|_2_|_3_|_4_|_5_|完全同意

7.产业参与可以帮助我熟悉工作。

完全不同意|_1_|_2_|_3_|_4_|_5_|完全同意

8.产业参与可以让我学习新知。

完全不同意|_1_|_2_|_3_|_4_|_5_|完全同意

9.产业参与可以帮助我提高工作任务完成效率。

完全不同意|_1_|_2_|_3_|_4_|_5_|完全同意

10.产业参与可以帮助我改善工作品质。

完全不同意|_1_|_2_|_3_|_4_|_5_|完全同意

11.我的朋友同事建议我参与产学研合作。

完全不同意|_1_|_2_|_3_|_4_|_5_|完全同意

12.学校高层建议我参与产学研合作。

完全不同意|_1_|_2_|_3_|_4_|_5_|完全同意

13.我的直属领导建议我参与产学研合作。

完全不同意|_1_|_2_|_3_|_4_|_5_|完全同意

14.我具有一定的专业领域知识储备。

完全不同意|_1_|_2_|_3_|_4_|_5_|完全同意

15.我具有较强的沟通协调能力。

完全不同意|_1_|_2_|_3_|_4_|_5_|完全同意

16.我具有一定的学术科研功底。

完全不同意|_1_|_2_|_3_|_4_|_5_|完全同意

17.我具有一定的专业技术水平。

完全不同意|_1_|_2_|_3_|_4_|_5_|完全同意

18.如果我尽力去做的话，我总是能够解决问题的。

完全不同意|_1_|_2_|_3_|_4_|_5_|完全同意

19.即使别人反对我，我仍有办法取得我所要的。

完全不同意|_1_|_2_|_3_|_4_|_5_|完全同意

20.如果我付出必要的努力，我一定能解决大多数难题。

完全不同意|_1_|_2_|_3_|_4_|_5_|完全同意

21.面对一个难题时，我通常能找到一些解决办法。

完全不同意|_1_|_2_|_3_|_4_|_5_|完全同意

22.不管有没有加班费，我都会将今日的工作完成。

完全不同意|_1_|_2_|_3_|_4_|_5_|完全同意

23.上班前，我会先想好今日应做工作清单与优先级。

完全不同意|_1_|_2_|_3_|_4_|_5_|完全同意

24.下班时，我清楚地知道今天交办事项与明日代办事项。

完全不同意|_1_|_2_|_3_|_4_|_5_|完全同意

25.我对自身的工作每周每月都有一定的排程。

完全不同意|_1_|_2_|_3_|_4_|_5_|完全同意

26.即使没有经济压力的情况下，我都还是会继续工作。

完全不同意|_1_|_2_|_3_|_4_|_5_|完全同意

27.我们学校的科研设备齐全、先进。

完全不同意|_1_|_2_|_3_|_4_|_5_|完全同意

28.我们学校具有完善、成熟的产学研合作平台。

完全不同意|_1_|_2_|_3_|_4_|_5_|完全同意

29.我们学校会及时迅速地提供有关产学研合作的信息资源。

完全不同意|_1_|_2_|_3_|_4_|_5_|完全同意

30.我们学校有一定数量的社会服务机构的支持与合作。

完全不同意|_1_|_2_|_3_|_4_|_5_|完全同意

31.我们学校鼓励教师们有创意有理想有想法。

完全不同意|_1_|_2_|_3_|_4_|_5_|完全同意

32.学校同仁做有新意的教学或研究活动会受到鼓励和表彰。

完全不同意|_1_|_2_|_3_|_4_|_5_|完全同意

33.学校领导重视具有应用性价值的研究。

完全不同意|_1_|_2_|_3_|_4_|_5_|完全同意

34.学校领导重视每一位教师的工作贡献及学术成果转化。

完全不同意|_1_|_2_|_3_|_4_|_5_|完全同意

35.学校教师们彼此会自由开放地交换看法。

完全不同意|_1_|_2_|_3_|_4_|_5_|完全同意

36.我相信我们学校不会做出伤害老师的事情。

完全不同意|_1_|_2_|_3_|_4_|_5_|完全同意

37.我相信老师遭遇难题时，学校会提供必要的协助。

完全不同意|_1_|_2_|_3_|_4_|_5_|完全同意

38.我相信校长会站在老师的立场替老师着想。

完全不同意|_1_|_2_|_3_|_4_|_5_|完全同意

39.我相信在我们学校工作会受到保障。

完全不同意|_1_|_2_|_3_|_4_|_5_|完全同意

40.我会尝试参与联合或合作研究（由企业提供经费）。

完全不同意|_1_|_2_|_3_|_4_|_5_|完全同意

41.我会尝试与产业界联合申报课题，共同研究政府资助项目。

完全不同意|_1_|_2_|_3_|_4_|_5_|完全同意

42.我会尝试为产业界提供技术咨询或技术服务。

完全不同意|_1_|_2_|_3_|_4_|_5_|完全同意

43.我会尝试带学生到企业实习或挂职锻炼。

完全不同意|_1_|_2_|_3_|_4_|_5_|完全同意

44.我会尝试与企业联合创办研究实体（如联合实验室、合作研究中心等）。

完全不同意|_1_|_2_|_3_|_4_|_5_|完全同意

45.我会参加由产业界举办的会议或论坛。

完全不同意|_1_|_2_|_3_|_4_|_5_|完全同意

到此，您已完成所有问题。非常感谢您的耐心参与，祝您工作顺利！
