# Supplementary material for: Impact of organizational culture, occupational commitment and industry-academy cooperation on vocational education in China: Cross-sectional Hierarchical Linear Modeling analysis
Source: PLoS One. 2022 Feb 23;17(2):e0264345. doi: 10.1371/journal.pone.0264345 (PMC8865651; doi:10.1371/journal.pone.0264345)
Supplement: S2 Questionnaire — (DOCX) [file pone.0264345.s002.docx]

**Survey Questionnaire of Teacher Industry Participation Behavior in Vocational Colleges**

Hello! This is a questionnaire survey conducted by the research team of the School of Public Administration of Sichuan University. The purpose is to understand the factors affecting the industrial participation of teachers and scholars in vocational colleges. Please tick "√" in the appropriate options according to the actual situation. It takes 5-10 minutes of your valuable time to fill out this questionnaire. We cherish and value your opinions very much. All the information you answer is for research purposes only. This research will comply with the 13th of the "Statistical Law of the People's Republic of China" According to Article 14 of the chapter, all the information you answer will be kept confidential. Please don't worry and answer with confidence. Thank you very much for your assistance and cooperation!

For the following descriptions, please tick "√" on the options that meet the situation:

1. Basic information

1. What is your gender?

(1) Male

(2) Female

2. Your job title?

(1) Teaching Assistant

(2) Lecturer

(3) Associate Professor

(4) Professor

3. What is your teaching age?

(1) Less than 5 years

(2) 5-10 years

(3) 10-15 years

(4) More than 15 years

4. Do you hold an administrative position?

(1) Yes

(2) No

5. The city and name of your school? (In order to facilitate the grouping, all teachers must fill in it, thank you very much!)

In the following description, please answer your own thoughts according to the prompt language of the question. The numbers 1 to 5 respectively represent the content of the team’s question completely disagree, disagree, general, agree, and completely agree. Please tick “√” under the score option that you think best represents your true thoughts according to the content of the answer:

2. Survey content

1. My stay in the academic field of vocational education is of great significance to me.

Disagree Completely|_1_|_2_|_3_|_4_|_5_|Completely agree

2. I must remain loyal to the vocational education work.

Disagree Completely|_1_|_2_|_3_|_4_|_5_|Completely agree

3. If I leave the vocational education job now, it will make me feel very empty.

Disagree Completely|_1_|_2_|_3_|_4_|_5_|Completely agree

4. After teachers receive vocational education and training, they should not change jobs at will.

Disagree completely|_1_|_2_|_3_|_4_|_5_|_Agree completely

5. Vocational education has helped a lot in shaping my personal image.

Disagree Completely|_1_|_2_|_3_|_4_|_5_|Completely agree

6. I like to accept and deal with the challenges of vocational education work.

Disagree Completely|_1_|_2_|_3_|_4_|_5_|Completely agree

7. Industry participation can help me get acquainted with my work.

Disagree Completely|_1_|_2_|_3_|_4_|_5_|Completely agree

8. Industry participation allows me to learn new knowledge.

Disagree Completely|_1_|_2_|_3_|_4_|_5_|Completely agree

9. Industry participation can help me improve the efficiency of completing work tasks.

Disagree Completely|_1_|_2_|_3_|_4_|_5_|Completely agree

10. Industry participation can help me improve the quality of my work.

Disagree Completely|_1_|_2_|_3_|_4_|_5_|Completely agree

11. My friends and colleagues suggested that I participate in industry-university-research cooperation.

Disagree Completely|_1_|_2_|_3_|_4_|_5_|Completely agree

12. The school senior suggested that I participate in industry-university-research cooperation.

Disagree Completely|_1_|_2_|_3_|_4_|_5_|Completely agree

13. My immediate leader suggested that I participate in industry-university-research cooperation.

Disagree Completely|_1_|_2_|_3_|_4_|_5_|Completely agree

14. I have a certain reserve of professional knowledge.

Disagree Completely|_1_|_2_|_3_|_4_|_5_|Completely agree

15. I have strong communication and coordination skills.

Disagree Completely|_1_|_2_|_3_|_4_|_5_|Completely agree

16. I have a certain degree of academic research skills.

Disagree Completely|_1_|_2_|_3_|_4_|_5_|Completely agree

17. I have a certain level of professional technology.

Disagree Completely|_1_|_2_|_3_|_4_|_5_|Completely agree

18. If I try my best, I can always solve the problem.

Disagree Completely|_1_|_2_|_3_|_4_|_5_|Completely agree

19. Even if others oppose me, I still have a way to get what I want.

Disagree Completely|_1_|_2_|_3_|_4_|_5_|Completely agree

20. If I put in the necessary efforts, I will be able to solve most problems.

Disagree Completely|_1_|_2_|_3_|_4_|_5_|Completely agree

21. When faced with a problem, I can usually find some solutions.

Disagree Completely|_1_|_2_|_3_|_4_|_5_|Completely agree

22. Regardless of whether there is overtime pay, I will finish today's work.

Disagree Completely|_1_|_2_|_3_|_4_|_5_|Completely agree

23. Before going to work, I will first think about the list and priority of the work to be done today.

Disagree Completely|_1_|_2_|_3_|_4_|_5_|Completely agree

24. When I get off work, I clearly know what to do today and what to do tomorrow.

Disagree Completely|_1_|_2_|_3_|_4_|_5_|Completely agree

25. I have a certain schedule for my work every week and every month.

Disagree Completely|_1_|_2_|_3_|_4_|_5_|Completely agree

26. Even if there is no financial pressure, I will continue to work.

Disagree Completely|_1_|_2_|_3_|_4_|_5_|Completely agree

27. The scientific research of our school is well-equipped and advanced.

Disagree Completely|_1_|_2_|_3_|_4_|_5_|Completely agree

28. Our school has a complete and mature industry-university-research cooperation platform.

Disagree Completely|_1_|_2_|_3_|_4_|_5_|Completely agree

29. Our school will promptly and quickly provide information resources related to industry-university-research cooperation.

Disagree Completely|_1_|_2_|_3_|_4_|_5_|Completely agree

30. Our school has the support and cooperation of a certain number of social service organizations.

Disagree Completely|_1_|_2_|_3_|_4_|_5_|Completely agree

31. Our school encourages teachers to have creativity, ideals and ideas.

Disagree Completely|_1_|_2_|_3_|_4_|_5_|Completely agree

32. School colleagues will be encouraged and commended for innovative teaching or research activities.

Disagree Completely|_1_|_2_|_3_|_4_|_5_|Completely agree

33. School leaders attach importance to research with applied value.

Disagree Completely|_1_|_2_|_3_|_4_|_5_|Completely agree

34. School leaders value the contribution of each teacher and the transformation of academic achievements.

Disagree Completely|_1_|_2_|_3_|_4_|_5_|Completely agree

35. School teachers will exchange views with each other freely and openly.

Disagree Completely|_1_|_2_|_3_|_4_|_5_|Completely agree

36. I believe that our school will not do anything to harm teachers.

Disagree Completely|_1_|_2_|_3_|_4_|_5_|Completely agree

37. I believe that the school will provide necessary assistance when teachers encounter problems.

Disagree Completely|_1_|_2_|_3_|_4_|_5_|Completely agree

38. I believe that the principal will stand in the teacher's position and think for the teacher.

Disagree Completely|_1_|_2_|_3_|_4_|_5_|Completely agree

39. I believe that working in our school will be guaranteed.

Disagree Completely|_1_|_2_|_3_|_4_|_5_|Completely agree

40. I will try to participate in joint or cooperative research (funded by the company).

Disagree Completely|_1_|_2_|_3_|_4_|_5_|Completely agree

41. I will try to jointly apply for projects with the industry and jointly study government-funded projects.

Disagree Completely|_1_|_2_|_3_|_4_|_5_|Completely agree

42. I will try to provide technical advice or technical services to the industry.

Disagree Completely|_1_|_2_|_3_|_4_|_5_|Completely agree

43. I will try to take students to the company for internships or on-the-job training.

Disagree Completely|_1_|_2_|_3_|_4_|_5_|Completely agree

44. I will try to co-found research entities with companies (such as joint laboratories, cooperative research centers, etc.).

Disagree Completely|_1_|_2_|_3_|_4_|_5_|Completely agree

45. I will participate in conferences or forums organized by the industry.

Disagree Completely|_1_|_2_|_3_|_4_|_5_|Completely agree

At this point, you have completed all questions. Thank you very much for your patience and participation, and I wish you success in your work!
